# Exploring the Potential Effectiveness of *Croton tiglium* Oil and Its Nano-Emulsion on *Earias insulana* (Lepidoptera: Nolidae)

**DOI:** 10.3390/insects16010072

**Published:** 2025-01-12

**Authors:** Karima S. Khater, Marwa M. Abd-Elrhmman, Zeinab M. E. A. Said, Ali A. El-Sayed, Abdelhadi A. I. Ali, Lamya Ahmed Alkeridis, Laila A. Al-Shuraym, Jingwen Wang, Qichun Zhang, Ahmed A. A. Aioub

**Affiliations:** 1 Zoology Department, Faculty of Science, Zagazig University, Zagazig 44511, Egypt; 2 Plant Protection Research Institute, Agricultural Research Center, Dokki, Giza 12618, Egypt; 3 Plant Protection Department, Faculty of Agriculture, Zagazig University, Zagazig 44511, Egypta.aioub@zu.edu.eg (A.A.A.A.); 4 Department of Biology, College of Science, Princess Nourah bint Abdulrahman University, P.O. Box 84428, Riyadh 11671, Saudi Arabia; 5 Agricultural Technology Extension Center of Hangzhou, Hangzhou 310058, China; 6 Zhejiang Provincial Key Laboratory of Agricultural Resources and Environment, Key Laboratory of Environment Remediation and Ecological Health, Zhejiang University, Ministry of Education, Hangzhou 310058, China

**Keywords:** plant extract oil, nano-emulsion, bioassay, histological examination, transmission electron microscope, *Earias insulana*

## Abstract

*Earias insulana Boisd.* (Lepidoptera: Nolidae) is a major pest of cotton and other crops in Egypt, and the widespread use of insecticides has led to resistance. This study evaluates, for the first time, the bioactivity of *Croton tiglium* (Malpighiales: Euphorbiaceae) oil and its nano-emulsion (CTNE) against 25 newly hatched larvae of *E. insulana* Boisd. We assessed their biological effects across different developmental stages and performed histological and ultrastructural examinations. Gas–liquid chromatography (GLC) identified several bioactive compounds in *C. tiglium* oil crushed dry seeds, including fatty acids, hydrocarbons, and sterols. CTNE showed excellent quality with a zeta potential of −17.7 mV, an average particle size of 54.28 nm, and spherical droplets of 42.42 nm in diameter. The LC_50_ values for *C. tiglium* oil and CTNE were 9.02% and 2.70%, respectively. Both treatments significantly impacted the biological characteristics of *E. insulana* Boisd., including reduced larval and pupal weight, lower adult emergence, decreased fecundity, and increased mortality. Histologically, there was epithelial cell hypotrophy and detachment, while ultrastructural damage included chromatin condensation, nuclear envelope folding, and mitochondrial damage, indicating apoptotic degeneration. These findings suggest *C. tiglium* oil and CTNE as potential, safe alternatives to chemical insecticides.

## Simple Summary

*Earias insulana Boisd.* (Lepidoptera: Nolidae) is a major pest affecting cotton and other crops in Egypt, with insecticide resistance becoming a growing issue. This study investigates the potential of *Croton tiglium* oil and its nano-emulsion (CTNE) as an alternative bioinsecticide. It is the first study to examine the effects of these treatments on newly hatched *E. insulana* larvae and their impact on various developmental stages of the pest. The analysis revealed that *C. tiglium* oil contains several bioactive compounds that are toxic to *E. insulana*. The nano-emulsion (CTNE) showed excellent properties with small particle size and stability. Both *C. tiglium* oil and CTNE proved highly toxic to the larvae, affecting their growth and development. These treatments led to reduced larval and pupal weight, lower adult emergence, decreased reproductive capacity, and increased mortality. Additionally, the treatments caused delays in larval and pupal development. Histological and ultrastructural studies revealed significant damage to the midgut cells, including cell shrinkage, vacuolation, and detachment. Nuclear changes, such as chromatin condensation and damaged mitochondria, were observed, indicating that the treatments caused cell death. Overall, the findings suggest that *C. tiglium* oil and its nano-emulsion could be an effective and safe alternative to chemical insecticides for controlling *E. insulana*.

## 1. Introduction

*Earias insulana* Boisd. spiny bollworm (Lepidoptera: Nolidae) is a critical pest attacking cotton crops in Egypt and causing great damage to cotton yield [1]. *Earias insulana* larvae feed on soft and growing parts of cotton, with a preference for the terminal bud, resulting in “top boring” damage before moving on to attack flower buds and bolls, which eventually drop off [2]. *Earias insulana* Boisd. causes bud shedding (12.5–16.6%), flower shedding (0.9–2.5%), and boll shedding (7.9–9.5%), culminating in 3.8–12.6% fruit damage in cotton crops [3]. This pest damages the crop during both vegetative and reproductive stages, leading to a substantial decline in yield [4]. The expansion of the use of pesticides to combat *E. insulana* has led to its resistance, reducing the effectiveness of pest control measures and necessitating the use of higher doses or alternative chemicals [5]. This over-reliance on insecticides has also contributed to environmental pollution, negatively impacting non-target organisms, soil health, and overall ecosystem balance [6]. The Recent European legislation (Reg. CE 396/2005, 1095/2007, 33/2008, 299/2008, and 1107/2009), which has imposed stricter limits on pesticide use in agriculture, has created a growing need to promote alternative pest control methods that are sustainable and economical. Therefore, innovative and low-cost approaches to control *E. insulana* are essential.

Biopesticides, originating from natural products such as plants, are eco-friendly, less toxic, and have less resistance build-up among insects and pathogens [7]. *Croton tiglium* L., belonging to the Euphorbiaceae family, thrives in tropical and sub-tropical regions [8]. Its leaves, seeds, stem, roots, and bark juice are prized globally for medicinal and dietary uses due to their rich essential oil content [9]. *Croton tiglium* is well-known for its pest toxicity due to active compounds in its seed oil, including phorbol esters, crotonic acid, and fatty acids [10]. Ahmed, et al. [11] reported that *C. tiglium* seed extracts influenced the tunneling behavior of *Odontotermes obesus*, disrupted commercial bacterial colony counts in their hindgut, and modified enzyme activity levels in their midgut. Another study demonstrated that *C. tiglium* seed and leaf extracts at 250 µg/mL were effective against *Aedes aegypti* larvae [12].

Nanotechnology offers the potential to enhance the efficacy and durability of active compounds, minimize agricultural inputs, and address the limitations of traditional pesticides [13,14]. The development of the nanotechnology process can improve its absorptivity, clarity, stability, and activity due to the small particle size, which makes the particle’s surface wider [15]. Notably, essential oil-based nano-emulsions are environmentally friendly and advantageous in contemporary agriculture and pest control [16]. Nano-emulsions are water-based, requiring significantly fewer organic solvents than conventional emulsion concentrates [17]. Additionally, Mushtaq, et al. [18] stated that nano-emulsions are an efficient strategy to increase the stability of the characteristics of bioactive materials, reduce volatility, and prevent environmental impacts. The application of nano-biopesticides for pest management is still limited, and information regarding their synthesis, variations, effectiveness, and mode of action is still lacking [19]. Therefore, optimizing the role of *C. tiglium* as a botanical insecticide against *E. insulana* can be done through technological development into nano-emulsion form.

This research hypothesizes that *C. tiglium* oil and its nano-emulsion can effectively control *E. insulana* while minimizing environmental risks associated with insecticide pollution. Our study aimed to identify the chemical compounds in *C. tiglium* oil using GLC, as well as the bioassay of *C. tiglium* oil and its nano-emulsion on the newly hatched *E. insulana* larvae, and their biological effects across various developmental stages. Furthermore, we also aimed to examine the histological and ultrastructure changes in the midgut of *E. insulana* larvae under *C. tiglium* oil and its nano-emulsion stress compared with untreated larvae.

## 2. Materials and Methods

### 2.1. Insect Rearing

A laboratory strain of *E. insulana* was sourced from a colony maintained at the Bollworms Research Department, Plant Protection Research Institute, Agricultural Research Center, Giza, Egypt. The strain had been reared on a semi-artificial diet in an incubator under controlled conditions: 26 ± 1 °C, 65 ± 5% RH, and a 12:12 (L:D) photoperiod for 10 generations without insecticide exposure [20].

### 2.2. Croton tiglium Oil Extraction

*Croton tiglium* fixed oil was sourced from the National Research Institute, Egypt. Briefly, *C. tiglium* fixed oil was extracted from 100 g of crushed dry seeds at room temperature according to Abdalaziz, et al. [21]. The seeds were immersed in petroleum ether as a solvent and filtered through anhydrous sodium sulfate. The solvent was eliminated using a rotary evaporator device; oils were kept in dark-brown bottles at 4 °C.

### 2.3. Analysis of C. tiglium Oil by Gas–Liquid Chromatography

*Croton tiglium* oil was analyzed using a Hewlett–Packard 6850 gas chromatograph according to Tarjan, et al. [22], where 0.2 µL of a solution containing essential oil and hexane were injected. Helium was used as the carrier gas, flowing at 0.7 mL/min. The analysis was performed on a DB-WAX capillary column, which was 20 m long, with an internal diameter of 0.1 mm and a stationary phase thickness of 0.25 µm. The oven temperature was initially set at 60 °C for 2 min, followed by a temperature increase of 12 °C/min until reaching 248 °C, which was held for 5 min. A flame ionization detector was employed for detection. Data were processed and compounds were identified using the National Institute of Standards and Technology (NIST) database through computer control.

### 2.4. Nano-Emulsion Preparation and Characterization

The nano-emulsion containing *C. tiglium* essential oil (CTNE) was synthesized at the National Research Center, Egypt following the method of El-Medany, et al. [23]. Briefly, O/W (oil-in-water) nano-emulsions were prepared by dissolving Tween 80 (3%, *v*/*v*) and ethanol (3%, *v*/*v*) in distilled water, which served as the aqueous phase. The oil phase was prepared by mixing *C. tiglium* essential oil (14%, *v*/*v* of the total coarse emulsion), representing 20% (*v*/*v*) of the overall emulsion, with the aqueous phase. The oil phase components were mixed thoroughly and then stored at 86 °C for 1 h. Afterward, the oil phase was combined with 80% water (*v*/*v*), with the addition of Tween 80 (3%, *v*/*v*) to stabilize the emulsion. The mixture was then stirred for 3 min and subjected to centrifugation at 10,000 rpm for 10 min to remove any large particles. The resulting nano-emulsion was stored in dark vials at room temperature until further analysis. The droplet size distribution of the *C. tiglium* oil nano-emulsion was characterized via dynamic light scattering (DLS), while the zeta potential and polydispersity index (PDI) were measured using photon correlation spectroscopy with a ZetaPlus tool (Malvern Zetasizer Nano-zs90, Malvern Instruments Ltd., Worcestershire, UK) [24].

### 2.5. Bioassay

Newly hatched first-instar *E. insulana* larvae were subject to 5, 7.5, 10, 12.5, 15, and 20% of *C. tiglium* oil and 1.25, 2.5, 5, and 10% of CTNE. These concentrations were determined following preliminary testing, where a range of concentrations was tested to identify effective but non-lethal levels for the larvae. Each concentration was sprayed on a Petri dish (8 cm diameter) and then filled with 5 g of artificial nutrition diet, and their mortality percentage was observed. Distilled water was utilized as a control. The newly hatched larvae were placed on each Petri dish. Each concentration and control have four replicates. Each replicate contains 25 freshly hatched larvae. The LC_25_, LC_50_, LC_75_, and LC_90_ values of *C. tiglium* oil and CTNE were calculated after 24 h, as well as a 95% confidence interval. Larvae that did not exhibit any response after touching with a brush were considered dead. The bioassay protocol was adapted from Ulhaq, et al. [25], with minor modifications to suit the experimental conditions.

### 2.6. Impact of C. tiglium Oil and Its Nano-Emulsion Sub-Lethal Exposure on E. insulana Different Development Stages

According to the bioassay results, surviving larvae from the treatments with the LC_50_ of *C. tiglium* oil and CTNE were transferred separately in 2 × 7 cm glass tubes with an untreated diet, covered with a piece of cotton, and maintained in the lab settings previously stated until pupation. Pupae were individually transferred into glass tubes until the moth emergency. After being sexed, just-emerged moths were placed in cages (two pairs per cage) and replicated four times. A honey solution (10%) was utilized to feed the moths. Larval and pupal stage period, mortality percentage and weight, pupation percent, the emergence of adult’s percentage, the lifespan, fecundity (number of eggs/female), hatching capacity percentage, and sex ratio were examined [26].

### 2.7. Histological and Ultrastructural Examination

Newly hatched larvae exposed to the LC_50_ concentration of *C. tiglium* oil and CTNE were placed on an untreated diet and kept under controlled laboratory conditions. Seven days post-treatment, necropsy samples were collected from both treated and control groups and then dissected to get the mid-gut of larvae. The samples were fixed in 10% formalin saline for 24 h, then rinsed with tap water and dehydrated using a graded series of ethyl alcohol solutions. After dehydration, the specimens were immersed in xylene for clearing and then embedded in paraffin for 24 h at 56 °C in a hot air oven. After preparing thin sections with a rotating LEITZ microtome, the specimens were deparaffinized and stained with hematoxylin and eosin. The stained tissue sections were mounted on glass slides for routine examination under a light microscope. The sections were thoroughly examined for histological changes [27]. Meanwhile, the ultrastructural impact of *C. tiglium* oil and CTNE on the midgut of both treated and control larvae was examined using transmission electron microscopy (TEM) [28].

### 2.8. Statistical Analysis

The corrected mortality percentages were analyzed statistically based on Abbott [29]. Also, Probit-MSChart software(Version 1.5) was used to determine the median lethal concentration values and 95% fiducial limits, following the methodology outlined by Finney [30] and using SPSS software (version 19.0, SPSS Inc., Chicago, IL, USA, 2003). The toxicity index was calculated according to Zidan and Abdel-Megeed [31] as:

Toxicity index = (LC_50_ of the most effective compound/LC_50_ of another tested compound) × 100.

One-way ANOVA analyzed the data obtained from a complete randomized design, with Duncan’s multiple-range test applied to compare the means at a significance level of *p* ≤ 0.05.

## 3. Results

### 3.1. Identification of C. tiglium Oil by Gas–Liquid Chromatography (GLC)

The GLC chromatogram of *C. tiglium* oil recorded 12 compounds of fatty acids by 100%, 8 compounds of hydrocarbon by 57.52%, and 6 compounds of sterols by 42.48%. Each phytocompound was identified according to its retention time, peak area, molecular weight, and molecular formula, as matched with the known compounds in the NIST library. The major compound of fatty acids in *C. tiglium* oil is linoleic acid (52.76%) followed by linolenic acid (16.85%). Meanwhile, docosane at 22.48% and tetracosane at 21.39% are the major compounds in hydrocarbon. Meanwhile, stigmasterol at 18.76 and β-sitosterol at 7.27% are compounds in sterols (Table 1).

### 3.2. Characterization of C. tiglium Oil Nano-Emulsion

**Figure 1:**
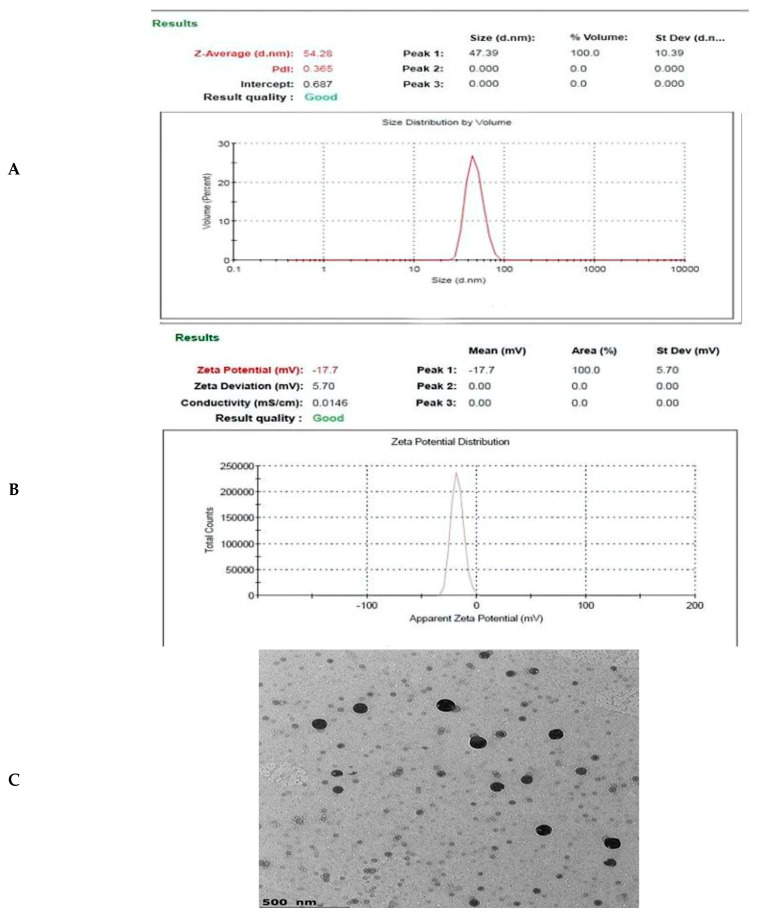
Characterization of *C. tiglium* nano-emulsion (CTNE) (**A**) DSL measurement size, (**B**) surface net negative charge by zeta potential (−17.7 mV), (**C**) TEM image observes the average size of spherical CTNE.

CTNE was prepared using high-pressure homogenization, which resulted in a mean droplet size ranging from 28.39 to 65.72 nm, expressed as the z-diameter. The polydispersity index was 0.365, indicating a uniform distribution of droplet sizes with minimal variation (Figure 1A). Moreover, the zeta potential of CTNE was relatively low, with an absolute value of −17.7 mV (Figure 1B). Moreover, TEM analysis revealed that the particle size was 42.42 nm. The droplets were spherical (Figure 1C).

### 3.3. Insecticidal Activity of C. tiglium Oil and Its Nano-Emulsion on E. insulana Newly Hatched Larvae

The bioassay results of *E. insulana* larvae showed a response to *C. tiglium* oil and its nano-emulsion after 1 day of treatment (Table 2). The LC_50_ and LC_90_ values were 9.02 and 18.60% for *C. tiglium* oil and 2.70 and 9.43% for CTNE, respectively.

### 3.4. Toxicological and Developmental Effects of C. tiglium Oil and Nano-Emulsion on E. insulana

Table 3, Table 4 and Table 5 show the influence of LC_50_ for *C. tiglium* oil and CTNE on the morphological characterization of *E. insulana* at different stages. The larvae and pupal duration of *E. insulana* were 17.91 and 12.17 days with *C. tiglium* oil treatment and 17.27 and 13.18 days with the CTNE treatment compared with the control at 12.83 and 9.01 days, respectively. Moreover, the larvae and pupal weight were significantly reduced from 0.094 and 0.071 g to 0.070 and 0.055 g with *C. tiglium* oil and 0.064 and 0.053 g with CTNE, respectively. The mortality percentages of *C. tiglium* oil and CTNE were recorded at 63.71 and 78.75% in *E. insulana* larvae and 19.93 and 22.74% in *E. insulana* pupal compared with the control, respectively (Table 3 and Table 4). In the adult stages (Table 5), CTNE significantly reduced the adult emergence percentage more than *C. tiglium* oil, compared with the control. In addition, the male and female longevity was 14.21 and 13.95 days with CTNE followed by 15.05 and 14.25 days with *C. tiglium* oil compared with the control. Interestingly, the sex ratios skewed towards males with *C. tiglium* oil and its nano-emulsion treatments at 59.84 and 64.63%, while the female percentage was 40.16% and 35.37% compared with the control, respectively. The pre-oviposition and post-oviposition periods were shorter with *C. tiglium* oil than with CTNE compared to the control. The oviposition periods remarkably decreased from 13.93 to 8.96 days with *C. tiglium* oil followed by CTNE (8.05 days). Furthermore, the number of eggs laid was 143.62 eggs, with a hatchability rate of 77.30% in *C. tiglium* oil treatment and 106.80 eggs with a hatchability rate of 66.37% in CTNE treatment compared with non-treated insects, respectively.

### 3.5. Histopathological Findings

**Figure 2:**
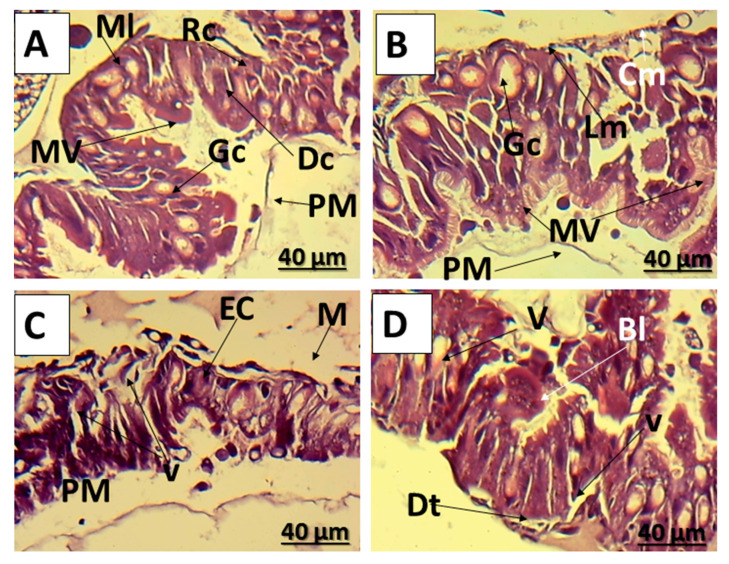
Photomicrographs of transverse section of midgut of *E. insulana* larvae showing, (**A**,**B**) normal midgut with layer of columnar epithelium, goblet cell, regenerative cell enclosed by layer of longitudinal and circular muscles; (**C**) larvae treated with LC_50_ of *C. tiglium* oil showing hypotrophy of epithelial cells; (**D**) larvae treated with LC_50_ of *C. tiglium* oil nano-emulsion showing, dislocation of epithelial cells away from its muscle layer, degenerated microvilli, large vacuole lack of peritrophic membrane and blebbing of cytoplasm (Bl: blebbing, CM: circular muscles, Dc, digestive cell; Dt: detachment, EC: epithelial cell, GC: goblet cell, LM: longitudinal muscles, ML: muscle layer, PM: peritrophic membrane, RC: regenerative cells and V: vacuole).

The histological structure of the midgut of untreated *E. insulana* 7-day-old larvae displayed typical anatomical features, including a foregut, midgut, and hindgut forming their alimentary canal. The midgut, noted for its efficiency in digestion and assimilation, stood out as particularly effective. Encasing the lumen was the peritrophic membrane. Within the midgut, a single layer of epithelial cells comprised two distinct types: goblet cells and columnar digestive cells, their quantities being roughly equivalent (Figure 2A). Digestive cells were noted for their discernible cytoplasm and distinctive nuclei housing condensed chromatin granules. Their exposed apical surface exhibited a distinct brush boundary, while the midgut lumen boasted a well-formed peritrophic membrane (Figure 2A,B). The nucleus was contemplated at the base, while the goblet cells were highly polarized. A layer of muscle made up of outer groups of longitudinal muscles and inner circular muscles encircled the basal plane of the epithelial cells (Figure 2A,B). On the other hand, the midgut of *E. insulana* larvae treated with LC_50_ of *C. tiglium* oil showed hypotrophy of epithelial cells, a vacuolated appearance, and degenerative indications (Figure 2C). The deformities increased with LC_50_ of CTNE, as observed in the midgut epithelial cells, which appeared highly disorganized. Cell borders were indistinct and detached from the muscular layer. Cytoplasmic vesicles were sparse, and the peritrophic membrane was absent. Furthermore, microvilli exhibited distortions in multiple areas (Figure 2D).

### 3.6. Ultrastructure Observations of Midgut Tissues of E. insulana under C. tiglium Oil and Its Nano-Emulsion Stress

**Figure 3:**
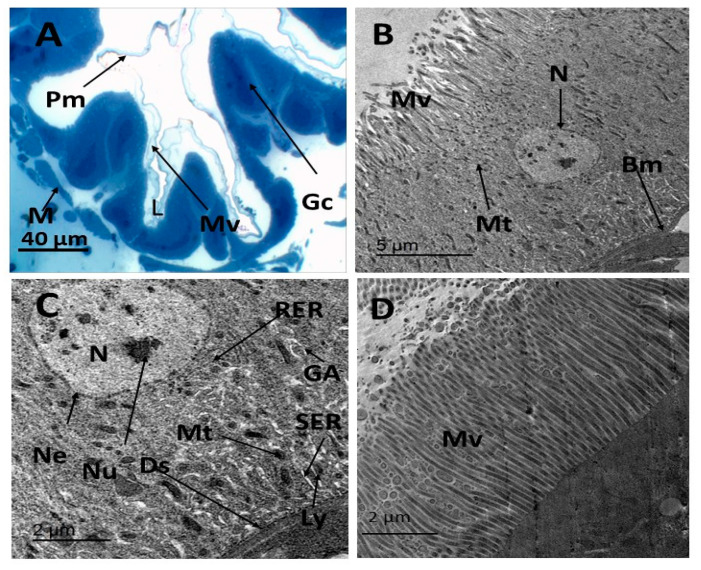
(**A**) A thin section in the midgut of the 7-day-old larvae of *E. insulana,* showing a single layer of columnar epithelium rests on basement membrane and peritrophic membrane (**B**–**D**) TEM photograph in the midgut of normal 7-day-old larvae of *E. insulana,* showing (**B**) epithelial cell has rounded nucleus with chromatin and prominent nucleolus, the cytoplasm has numerous mitochondria and microvilli at free border (**C**) the nucleus with the clear nuclear envelope, rough endoplasmic reticulum with ribosomes, smooth endoplasmic reticulum, Golgi apparatus, and lysosomes and desmosomes between two adjacent cells; (**D**) elongated microvilli at the apical free border. (Bm, basement membrane; Ch, chromatin; De, desmosome; Ga, Golgi apparatus; Gc, gastric cell; Ly, lysosomes; M, muscles; Mt, mitochondria; Mv, microvilli; N, nucleus; Ne, nuclear envelop; Nu, nucleolus; Pm, peritrophic membrane; RER, rough endoplasmic reticulum; and SER, smooth endoplasmic reticulum).

Electron micrographs of the midgut of *E. insulana* in the untreated group revealed a single layer of epithelial cells resting on a continuous basement membrane. A brush border of microvilli lined the lumen, while a layer of muscle encircled the basal plane of the epithelial cells (Figure 3A). The midgut epithelium contained centrally positioned nuclei with uncondensed chromatin, surrounded by a distinct nuclear membrane. Numerous round and elongated mitochondria were observed, clustered near the apical region of the cells (Figure 3B). This arrangement facilitates active transport. The presence of Golgi cristae suggested a prominent secretory function of the cells (Figure 3B). The cytoplasm was notably rich in the endoplasmic reticulum, with the rough endoplasmic reticulum frequently organized into complex stacks (Figure 3C). Lysosomes were dispersed throughout the cytoplasm, and smooth endoplasmic reticulum was also visible. The plasma membrane was often integrated with desmosomes for adhesion to neighboring cells (Figure 3C). The apical free border featured elongated microvilli (Figure 3D), further emphasizing the structural specialization of the epithelium.

**Figure 4:**
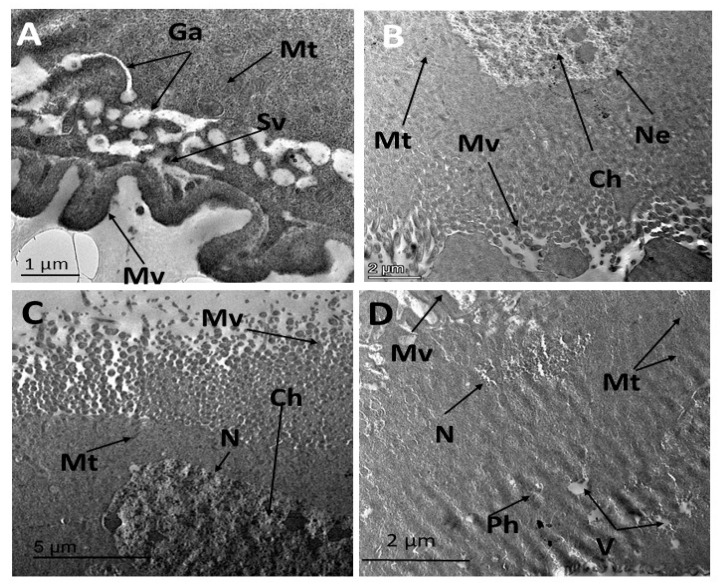
(**A**) TEM photograph of normal midgut cell showing a free border with numerous elongated microvilli, vesicles of Golgi system, fat vacuoles, and rounded-shaped mitochondria; (**B**–**D**) TEM photograph in midgut of 7-day-old larvae of *E. insulana* treated with LC_50_ of *C. tilgium* oil showing, (**B**,**C**) folded nuclear envelop, condensed chromatin material, and distorted mitochondria, (**D**) apoptic nucleus with undefined nuclear envelop, vacuolation in cytoplasm, degenerated mitochondria, and phagolysosome (Ch, chromatin; Ga, Golgi apparatus; Gc, gastric cell; M, muscles; Mt, mitochondria; Mv, microvilli; Ne, nuclear envelop; Pm, peritrophic membrane; Sg, secretory granule; Sv, secretory vesicles).

**Figure 5:**
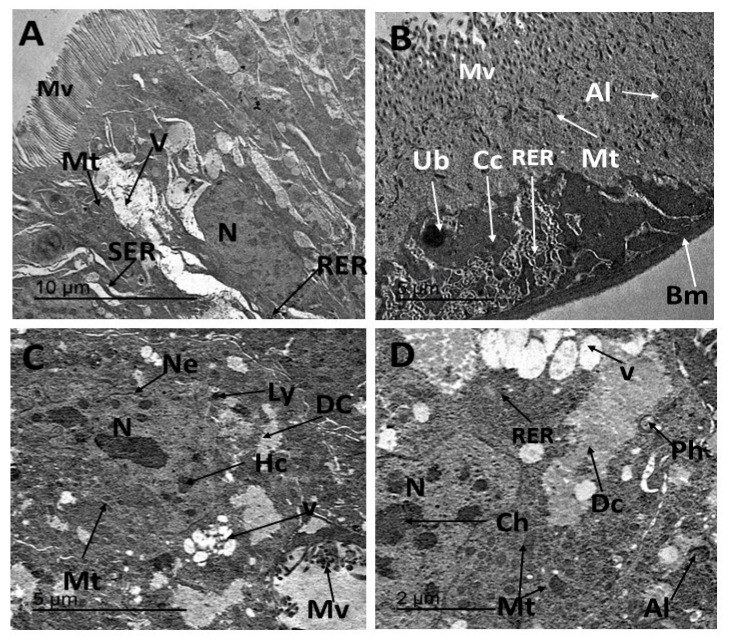
(**A**) TEM photographs in midgut of 7-day-old larvae of *E. insulana* treated with LC_50_ of nano-emulsion of *Croton tilgium* showing, (**A**) cytoplasm vacuolation, enlargement of mitochondria, fragmentation in RER, nuclear atrophy, and presence of heterochromatin patches; (**B**) strong condensed cytoplasm, undigested body, degenerated RER, distorted mitochondria, and autolysosome; (**C**) strong folded nuclear envelope, degenerated cytoplasm, lysosome, numerous vacuoles, distorted mitochondria and microvilli, and lysosome; (**D**) dilated mitochondria, folded nuclear envelope, large vacuoles, autolysosome, and phagosome. (Al, autolysosome; Ch, chromatin; Dc, degenerated cytoplasm; Mt, mitochondria; Ly, lysosome; Mv, microvilli; Ne, nuclear envelop; Ph, phagosome; RER, rough endoplasmic reticulum; and SER, smooth endoplasmic reticulum; V, vacuole).

Vesicles secreted from the Golgi complex were transported to the apex of the cell and fused with the apical plasma membrane near the base of the microvilli (Figure 4A). The ultrastructure of the midgut in *E. insulana* treated with the LC_50_ concentration of *C. tiglium* oil exhibited clear and significant pathological alterations. Nuclear changes included chromatin condensation, an early marker of apoptosis, and nuclear envelope folding. Distorted microvilli were also evident following treatment (Figure 4B,C). Cytoplasmic condensation was observed, along with chromatin aggregates that were not fully enclosed by the nuclear envelope. Phagolysosomes and cytoplasmic vacuolation indicated apoptotic degeneration, accompanied by distorted mitochondria (Figure 4D). Similarly, treatment with the LC_50_ concentration of CTNE induced pronounced ultrastructural abnormalities in midgut cells. These included degenerated mitochondria and pyknotic nuclei containing irregularly condensed chromatin. Cytoplasmic vacuolation and fragmentation of the rough endoplasmic reticulum (RER) were apparent, indicating necrotic degeneration. Swelling of the smooth endoplasmic reticulum was also observed (Figure 5A). Electron micrographs revealed condensed cytoplasm, large undigested bodies, degenerated RER, distorted mitochondria, and autolysosomes. Autophagic vacuoles and degradation of the RER were prominent (Figure 5B). Additional abnormalities included nuclear atrophy and small aggregates of condensed chromatin (Figure 5A,C). The nuclear envelope was strongly folded (Figure 5C,D). These changes were considered key cytopathological markers of apoptosis. Degenerated cytoplasm, lysosomes, numerous vacuoles, distorted mitochondria, and damaged microvilli were also detected (Figure 5C). Further evidence of apoptosis following CTNE treatment included mitochondrial dilation, nuclear envelope folding, and the presence of large vacuoles. Autolysosomes and phagosomes, indicative of detoxification through foreign substance degradation, were also observed (Figure 5D).

## 4. Discussion

The use of synthetic pesticides against this insect not only has led to increased resistance of the insect [32] but also impacts on ecosystems and non-target organisms [33]. Therefore, the shift towards plant-based insecticides from synthetic ones offers a promising avenue for efficient insect pest management and sustainable agriculture, driven by the identification of naturally occurring phytochemicals as active ingredients in botanical insecticides [34,35]. Our GLC study demonstrated that *C. tiglium* oil has several compounds, including 12 compounds of free fatty acids, 8 compounds of hydrocarbons, and 6 compounds of sterols. Linoleic acid (free fatty acids), docosane, tetracosane (hydrocarbons), and stigmasterol (sterols) compounds represent the major compounds in *C. tiglium* oil. Our findings are consistent with the research conducted by Shanmugapriya, et al. [36] that reported that *C. tiglium* seeds contained saturated fatty acids, including behenic acid and palmitic acid, as well as unsaturated fatty acids, including oleic acid and linoleic acid. Similarly, the authors found the major compounds in *C. tiglium* seeds were linoleic acid and stigmasterol [37]. Moreover, gas chromatography–mass spectrometry (GC-MS) analysis revealed some compounds in *C. tiglium* oil, including linoleic acid and oleic acid, which together accounted for 77.33% of the total oil in the ethyl–esterified sample [38].

Nano-emulsions, typically ranging from 20 to 500 nm in size, are also referred to as mini-emulsions, submicron emulsions, or ultra-fine emulsions [39]. The three main types of nano-emulsions are oil in water (O/W), and water in oil (W/O) [40]. It has been shown in earlier studies that nano-emulsions can also be toxic to insects [41,42]. The CTNE showed a conductivity value, indicating the presence of slightly conductive ions, which could prevent electrode polarization and enhance its stability and resistance to deterioration [43]. Also, the droplet size of *C. tiglium* nano-emulsions was 54.28 nm. These results are confirmed with Sugumar, et al. [44], which found that the ideal droplet size for a good nano-emulsion is between 20 and 200 nm. Furthermore, Wahba, Abdelatef, and Wahba [10] recorded that the DLS of the eugenol oil nano-emulsion was 54.68 nm, whereas the PDI was 0.365 and the formulation’s size-district zeta potentials of *C. tiglium* nano-emulsion had a relatively low absolute zeta potential of −17.7 mV. These values suggest that the nano-emulsion exhibited good physical stability, as Ostwald ripening was minimized [45]. The low viscosity, likely due to the reduced oil content, delayed instability processes and resulted in oil droplets with a more uniform particle size [46]. Our result corroborates the findings of Draz, et al. [47], which demonstrated that the zeta potentials of nano-emulsions of anise and thyme oils were between −14.5 and −27.8 mV, with PDI ranging from 0.209 to 0.37. Also, El-Naby, et al. [48] found that the nano-emulsion exhibited a zeta potential of −20 mV, a size distribution of 174.6 nm, and a PDI of 0.393. TEM analysis revealed that the droplets were spherical, with a particle size of 42.42 nm, which was consistent with the results from dynamic light scattering. Our results support the conclusions of Aioub, et al. [49], who reported that the morphology of cinnamon nano-emulsions (CMNEs) was spherical, with a particle size between 22–37 nm. Additionally, the spherical-form particles of *Mentha pulegium* nano-emulsion had a diameter range of 41–137 nm [23].

The spiny bollworm, *E. insulana*, has been recognized as one of the world’s most significant pests. It caused severe damage, resulting in a substantial decline in crop production [1]. Our study evaluated the toxicity of *C. tiglium* oil and its nano-emulsion against the different development stages of *E. insulana*. The LC_50_ values for *C. tiglium* oil and its nano-emulsion on the newly hatched larvae of *E. insulana* were 9.02 and 2.70%, respectively. This result was congruent with the findings of Eldesouky, et al. [50], who reported that the oleic and linoleic acids exhibit insecticidal effects against *Spodoptera littoralis* larvae. Additionally, β-sitosterol induces growth inhibition and systemic toxicity in *Helecoverpa armigera* [51]. Kannan, et al. [52] recorded that nano-materials are effective and eco-friendly bio-insecticides for pest control. Notably, The toxicity of three fatty acids (oleic, stearic, and linoleic acids) was observed under laboratory conditions against the first instar larvae of *E. insulana* [53]. The pepper plant essential oil has toxicity effects on the newly hatched larvae of *E. insulana* [54]. Al Shater, et al. [55] reported that silver nanoparticles (AgNPs) exhibited significant toxic effects against the cotton spiny bollworm, *E. insulana*, suggesting that AgNP treatment may be more effective than plant extracts alone for its control. In a study by Moustafa, et al. [56], the nano-emulsion of *Eucalyptus globulus* essential oil was tested on larvae of *E. insulana* and *Pectinophora gossypiella*, showing remarkable effectiveness in suppressing cotton bollworms.

The oil of *C. tiglium* and its nano-emulsion significantly increased mortality rates in the larval, pupal, and adult stages of *E. insulana*. Additionally, they prolonged the developmental durations of these stages, reduced larval and pupal weights, and decreased egg hatchability percentages. Furthermore, the oil and its nano-emulsion shortened the lifespans of both male and female moths, altered the sex ratio, and affected reproductive parameters. Specifically, they resulted in shorter oviposition and post-oviposition periods, as well as a longer pre-oviposition period compared to the control. Our results align with those of Eldesouky, Khamis, and Hassan [50], who reported a reduction in the pupal weight, pupation, adult emergence percentages, fecundity, and adult longevity of *S. littoralis* larvae treated with oleic and linoleic acids. Moreover, the higher prepupal and pupal mortality of *Helicoverpa armigera* compared to larval mortality, along with a greater reduction in adult emergence and the average weight gained by later instars, highlights the cumulative effects of β-sitosterol [51]. Furthermore, *C. tiglium* ethanol extracts had strong antifeedant activity and growth inhibition activity against *Plutella xylostella* larvae [57]. El-Din, et al. [58] reported that the LC_50_ values of jojoba oil and flaxseed oil for *E. insulana* significantly increased larval mortality and prolonged both larval and pupal durations. Additionally, these oils extended the pre-oviposition period while reducing adult emergence rates, the lifespan of males and females, the number of eggs laid, hatchability percentages, and the oviposition period. Another study found that treatment with gelatin-copper nanoparticles resulted in the highest larval and pupal mortality rates in *E. insulana* [59]. Similarly, Frankincense nano-emulsion negatively affected *Earias insulana* by reducing larval and pupal weights, prolonging larval development, and decreasing egg deposition, hatchability, and moth emergence [26]. El-Medany, El-Shennawy, and Kandil [23] demonstrated that *M. pulegium* nano-emulsion increased the percent of larval and pupal mortality and malformation, as well as the elongation of the larval period and pupal duration for *E. insulana*. All the above-mentioned examples supported the results obtained from the current study that *C. tiglium* oil and its nano-emulsion can be used for controlling the different development stages of *E. insulana*.

The efficacy of *C. tiglium* oil and its nano-emulsion against insects can be attributed to its unique chemical composition, which includes potent bioactive compounds with insecticidal and growth-regulating properties. These compounds, such as oleic, linoleic acids, palmitic acid, and β-sitosterol, caused disrupt various physiological processes in insects, including prolonged developmental durations, high mortality percentages, and decreased egg hatchability percentages [60]. Moreover, the prolongation of the larval and pupal stages could be attributed to the treatment’s interaction with the midgut epithelium, potentially disrupting the digestive system, which may also impact the pupation rate. *Croton tiglium* oil is thought to be a rich source of physiologically active substances, which act as feeding repellents that hinder the growth and survival of larvae [61]. Consequently, the malformation of pupae and adults in *Spodoptera litura* could be attributed to the toxicant’s effect during metamorphosis [62]. In addition, plant essential in the form of nano-emulsion is more toxic than plant essential oil on the influence of physiological processes in insects due to improved solubility, stability, and penetration into the insect’s cuticle, making it more effective at lower concentrations [63,64].

The histopathological effects of *C. tiglium* oil and its nano-emulsion on 7-day-old larvae of *E. insulana*, as observed in the current study, revealed severe disorganization of the midgut epithelial cells, with cell boundaries disappearing and detaching from the muscle layer. The peritrophic membrane was indistinct, and numerous cytoplasmic vesicles were observed, distorting the microvilli in many regions. Our results agreed with El-Medany, El-Shennawy, and Kandil [23], whose study on *E. insulana* larvae treated with *M. pulegium* oil nano-emulsion showed vacuolization and disorganization in the epidermal layer, along with poorly defined cuticular layers and scattered spines.

Our ultrastructural analysis showed that treatment with *Croton tiglium* oil revealed nuclear alterations, including chromatin clumping and nuclear envelope folding. Moreover, treatment with *C. tiglium* nano-emulsion induced pronounced ultrastructural abnormalities in midgut cells, characterized by severe folding of the nuclear membrane, chromatin clumping, and fragmentation of the RER. Autophagic vacuoles, indicative of apoptosis, were observed, along with cytoplasmic degeneration, a hallmark of apoptotic cytopathology. Additionally, distorted microvilli were prominently noted.

The damaging effect of *C. tiglium* oil and its nano-emulsion by histopathological and ultrastructural analysis may be due to fatty acids acting on the insect’s nervous system. The compounds need to traverse the cuticle, blood barrier, and perineurium of the insect. They destroy the fundamental unit of the nervous system, disrupting the insect’s behavior, movement, and other functions, ultimately leading to larval poisoning and death [65]. The authors found that fatty acids exhibited insecticidal effects against *Culex quinquefasciatus*, and histological examination of oleic and linoleic acids revealed their ability to induce cell instability in the midgut cells [66]. Moreover, the nano-emulsion leads to intensification in the permeability of the inner mitochondrial membrane, which in turn produces enlargement of the mitochondria, and, finally, cellular apoptosis [67]. As far as we are aware, no research has been conducted on the influence of *C. tilgium* oil and its nano-emulsion on the histopathological and ultrastructure of the midgut larvae of *E. insulana*.

Overall, our findings strongly suggest that *C. tiglium* oil and its nano-emulsion are effective in controlling *E. insulana*, overcoming resistance to synthetic pesticides, and promoting environmental protection by reducing pollution.

## 5. Conclusions

Managing *E. insulana* is crucial for agricultural sustainability, but the traditional reliance on chemical pesticides poses significant risks to human health and the environment. Therefore, our study focused on *C. tiglium* oil and its nano-emulsion as a biopesticide for controlling *E. insulana*. *Croton tiglium* oil in the form of nano-emulsion has proven to be more toxic than *C. tiglium* oil in the different development stages of *E. insulana* through some parameters, including bioassay experiments, biological effects, and histological and ultrastructure studies. Nano-formulated plant extracts exhibit enhanced efficacy against pests while minimizing environmental contamination. This approach underscores a shift towards sustainable agriculture, safeguarding ecosystems and promoting healthier farming practices for future generations. Future research should focus on investigating the effects of nano-emulsions on non-target organisms, particularly beneficial parasitoids and predators, to assess their safety and ensure ecological balance. Additionally, optimizing nano-formulations of plant extracts and essential oils for more effective pest control, while reducing toxicity to non-target species, and evaluating the susceptibility of other economically important pests to nano-emulsions, will expand the potential applications of these formulations in integrated pest management strategies.

## Figures and Tables

**Table 1:** Gas–liquid chromatography (GLC) analysis of *C. tiglium* oil.

No.	RT ^a^	Conc. (%)	Compound	Molecular Formula
Free fatty acids				
1	4.137	0.27	Methyl octanoate	C_9_H_18_O_2_
3	5.531	0.24	Methyl decanoate	C_12_H_24_O_2_
4	6.635	0.56	Lauric acid	C_12_H_24_O_2_
5	8.125	2.51	Tridecanoate	CH_3_(CH_2_)_11_COOH
6	9.693	2.97	Myristic acid	C_14_H_28_O_2_
7	10.265	2.17	Methyl Pentadecanoate	C_16_H_32_O_2_
8	11.921	5.89	Palmitic acid	C_16_H_32_O_2_
9	15.010	8.79	Oleic acid	C_18_H_34_O_2_
10	18.02	52.76	Linoleic acid	C_18_H_32_O_2_
11	20.22	16.85	Linolenic acid	C_18_H_30_O_2_
12	21.95	6.99	Behenic acid	C_22_H_44_O_2_
Hydrocarbon				
1	8.974	0.45	Pentadecane	C_15_H_32_
2	10.46	0.36	Hexadecane	C_16_H_34_
3	13.24	1.55	Octadecane	C_18_H_38_
4	14.62	2.49	Nonadecane	C_19_H_40_
5	16.95	3.67	Heneicosane	C_21_H_44_
6	18.79	22.48	Docosane	C_22_H_46_
7	20.93	5.13	Tricosane	C_23_H_48_
8	22.26	21.39	Tetracosane	C_24_H_50_
Sterols				
1	24.02	5.54	Squalene	C_30_H_50_
2	25.21	4.86	Cholesterol	C_27_H_46_O
3	25.70	4.49	Campasterol	C_28_H_48_O
4	26.86	18.76	Stigmasterol	C_29_H_48_O
5	29.42	7.27	β-sitosterol	C_29_H_50_O
6	32.73	1.56	α-amyrin	C_30_H_50_O

^a^ RT: Retention time.

**Table 2:** Toxicity data of *C. tiglium* oil and its nano-emulsion on the newly hatched larvae of the *E. insulana* after 1-day treatment.

Treatments	Lethal Concentration Value (%)	Conc. %	Fiducial Limits (95%)		Slope	Toxicity Index LC_50_ %	*p*-Value	X^2^
			Lower	Upper				
*C. tiglium* oil	LC_25_	6.15	3.31	6.72	4.05 ± 0.38	29.93	0.436	2.724
	LC_50_	9.02	6.59	11.6				
	LC_75_	13.2	11.7	22.6				
	LC_90_	18.6	18.3	66.8				
NE of *C. tiglium* oil (CTNE)	LC_25_	1.31	1.01	1.61	2.16 ± 0.22	100	0.232	4.289
	LC_50_	2.70	2.30	3.12				
	LC_75_	5.55	4.71	6.84				
	LC_90_	9.43	8.33	14.8				

X^2^: chi-square goodness-of-fit statistic.

**Table 3:** Effect of medium lethal concentration of *C. tiglium* oil and its nano-emulsion on *E. insulana*.

Treatment	Larval Duration (Day)	Larval Weight (g)	Laval Mortality (%)
*C. tiglium* oil	17.91 ^a^ ± 0.187	0.070 ^b^ ± 0.001	63.71 ^b^ ± 0.425
NE of *C. tiglium* oil	17.27 ^b^ ± 0.102	0.064 ^c^ ± 0.001	78.72 ^a^ ± 0.557
Control	12.83 ^c^ ± 0.155	0.094 ^a^ ± 0.001	3.470 ^b^ ± 0.105
*p* values	0.007	0.024	0.015
*F* ratio	10.241	6.146	8.014

Each number represents the mean ± SE of four independent experiments. Different letters in each column indicate significant differences (*p* ≤ 0.05).

**Table 4:** Effect of medium lethal concentrations of *C. tiglium* oil and its nano-emulsion in the pupal stage of the *E. insulana* after larval exposure.

Treatment	Pupation (%)	Pupal Duration (day)	Pupal Weight (g)	Pupal Mortality (%)
*C. tiglium* oil	36.29 ^b^ ± 0.425	12.17 ^b^ ± 0.129	0.055 ^b^ ± 0.001	19.93 ^b^ ± 0.563
NE of *C. tiglium* oil	21.28 ^c^ ± 0.251	13.18 ^a^ ± 0.116	0.053 ^b^ ± 0.001	22.74 ^a^ ± 0.340
Control	96.52 ^a^ ± 0.105	9.010 ^c^ ± 0.228	0.071 ^a^ ± 0.001	1.360 ^c^ ± 0.114
*p* values	0.004	0.008	0.042	0.001
*F* ratio	12.511	11.742	3.334	19.641

Each number represents the mean ± SE of four independent experiments. Different letters in each column indicate significant differences (*p* ≤ 0.05).

**Table 5:** Effect of medium lethal concentration of *C. tiglium* oil and its nano-emulsion on mature stage of the *E. insulana* resulted from larval treatment.

Treatments	Adult Emergence (%)	Female Longevity (Day)	Male Longevity (Day)	Sex Ratio		Pre-Oviposition Period	Oviposition Period	Post-Oviposition Period	Laid Eggs Number	Hatchability (%)
				♀	♂					
*C. tiglium* oil	80.07 ^b^ ± 0.563	14.25 ^b^ ± 0.276	15.05 ^b^ ± 0.309	40.16 ^b^ ± 0.988	59.84 ^b^ ± 0.988	3.110 ^a^ ± 0.108	8.960 ^b^ ± 0.272	2.180 ^b^ ± 0.095	143.6 ^b^ ± 2.553	77.30 ^b^ ± 0.357
NE of *C. tiglium* oil	77.26 ^c^ ± 0.340	13.95 ^b^ ± 0.403	14.21 ^c^ ± 0.208	35.37 ^c^ ± 0.316	64.63 ^a^ ± 0.316	3.430 ^a^ ± 0.139	8.050 ^c^ ± 0.221	2.470 ^ab^ ± 0.105	106.8 ^c^ ± 1.754	66.37 ^c^ ± 0.946
Control	98.64 ^a^ ± 0.114	18.73 ^a^ ± 0.102	17.31 ^a^ ± 0.119	50.31 ^a^ ± 0.312	49.69 ^c^ ± 0.312	1.870 ^b^ ± 0.161	13.93 ^a^ ± 0.257	2.930 ^a^ ± 0.213	252.0 ^a^ ± 3.628	96.31 ^a^ ± 0.373
*p*-values	0.013	0.040	0.018	0.011	0.010	0.039	0.021	0.044	0.002	0.006
*F* ratio	7.423	3.405	7.930	7.219	8.105	3.910	6.443	5.892	18.12	17.29

Each number represents the mean ± SEM of four independent experiments. Different letters in the same column indicate significant differences (*p* ≤ 0.05).

## Data Availability

All data and materials are included in the manuscript.
